# Influence of lifestyle on postmenopausal women’s sleep

**DOI:** 10.1051/bmdcn/2018080313

**Published:** 2018-08-24

**Authors:** Zaira Fernanda Martinho Nicolau, Sergio Tufik, Helena Hachul

**Affiliations:** 1 Department of Ophthalmology, Federal University of São Paulo SP 04021-002 São Paulo Brazil; 2 Department of Psychobiology, Federal University of São Paulo SP 04021-002 São Paulo Brazil

The study of Moudi *et al* [1] highlights the influence of lifestyle on sleep quality in Iranian postmenopausal women. The authors emphasize that demographic variations are responsible for different results worldwide. We agree with this statement and would like to share and compare results from our center, located in Sao Paulo, Brazil.

The influence of exercises on sleep is noteworthy. For instance, post- menopause women practicing yoga have less insomnia complaints [2]. This can be due to an interaction with the neuroendocrine and autonomic nervous systems, which lead to improved sleep patterns and less vasomotor symptoms [3]. Nevertheless, patients practicing passive-stretching exercises did not have significant improvement [2].

There is also an influence of therapeutic massage and acupuncture in sleep architecture. Patients undergoing these interventions have a higher percentage of the N3 and N4 stages of sleep [4–6]. Massage also improves sleep efficiency [5] and well being upon awakening [6].

Regarding constitutional characteristics, obese women have more obstructive sleep apnea and difficulty to reach rapid eye movement (REM) stage [7]. A deep sleep is important for memory and body and mind restitution [8]. They also have a tendency of eye problems, such as floppy eyelid syndrome (FES) and discomfort on waking. There is an association between FES and sleeping posture and laterality adopted by the patient [9].

We would also like to point out that the amount of years after the menopause is an important factor to take in consideration. We have observed that patients in late post-menopause have more subjective complaints related to sleep, such as daytime sleepiness [10].

Postmenopausal women have more sleep disturbances than younger individuals [10]. The knowledge of lifestyle factors that influence sleep is important to afford a complete therapeutic management of this population.

## Authors’ contributions

The authors are equally responsible for the concept of this letter and for drafting the manuscript. The final version of the manuscript has been read and approved by all of the listed authors, who have each provided the attention necessary to ensure the integrity of the work.

## Funding/Support

Our studies are supported by grants conceived by AFIP, FAPESP and CNPq. The funding agencies had no role on design, preparation, review or approval of this letter.

## Conflict of interests

The authors declare that there are no financial or other relationships that might lead to conflicts of interest.
